# Parent–Child Associations in Accelerometer-Measured Physical Activity and Sedentary Behaviour: The FAMIPASS Study

**DOI:** 10.3390/children11060710

**Published:** 2024-06-08

**Authors:** Dagmar Sigmundová, Jaroslava Voráčová, Jan Dygrýn, Michal Vorlíček, Erik Sigmund

**Affiliations:** 1 Institute of Active Lifestyle, Faculty of Physical Culture, Palacký University Olomouc, 771 11 Olomouc, Czech Republic; jan.dygryn@upol.cz (J.D.); michal.vorlicek@upol.cz (M.V.); erik.sigmund@upol.cz (E.S.); 2 Department of Social Sciences in Kinanthropology, Faculty of Physical Culture, Palacký University Olomouc, 771 11 Olomouc, Czech Republic; jaroslava.voracova@upol.cz

**Keywords:** children, parents, physical activity, sedentary, ActiGraph

## Abstract

Parent–child patterns in objectively measured movement behaviours were the highlight of this study. A total of 381 families (337 mothers, 256 fathers, 190 daughters, and 191 sons) from 36 randomly selected schools and kindergartens provided valid accelerometer data. Sedentary behaviour and physical activity (PA) were assessed using ActiGraph accelerometers. Spearman’s rho was used to evaluate parent–child associations, while logistic regression analysis (the backward LR method) was used to recognize factors related to children’s achievement of PA recommendations. Results indicated that girls engaged more in light PA, while boys showed higher levels of moderate and vigorous PA. Mothers spent less time sitting and more time in light PA compared to fathers, resulting in higher total PA levels. Father–son pairs showed a stronger association in total PA than mother–son pairs. Children aged 6–10 years and those with mothers who engaged in more vigorous PA were more likely to meet PA recommendations compared to younger children and those with less active mothers.

## 1. Introduction

Despite the many health benefits of sufficient physical activity (PA) and reduced sedentary behaviour (SB) [1], a large number of youths still do not meet the recommended levels of PA [2]. This trend extends to preschoolers, as evidenced by a European study involving six countries (Belgium, Bulgaria, Germany, Greece, Poland, and Spain) [3]. Several factors influence the development and persistence of these movement behaviours in children [4,5]. Among them, the family plays a crucial role [4,5]. The quality of the parent–child association and parental behaviours, including modelling and support, profoundly impacts the health and behaviours of their children [4,5,6]. Associations between parents and children have been documented in PA and SB [7]. Mothers, compared to fathers, tend to play a greater role in modelling healthy active habits, whereas both parents equally influence children’s unhealthy SB [8].

A recent systematic review found a weak positive relationship in the PA of parent–child dyads. Similar findings were observed in gender-specific sub-groups, including father–child and mother–child pairs [7]. The strong PA parent–child association was also confirmed in 78% of studies from a systematic review (2001–2020) examining 6–12-year-old children and their parents [9]. However, research is limited on the number of studies examining father and child PA associations [10]. Since the majority of the studies on preschoolers and school-aged children included mother–child dyads, more studies should focus on recruiting male parents [7,11,12,13,14]. Moderate-to-vigorous PA (MVPA) has been used as the PA outcome variable in most of the studies, followed by total PA [7,12,13,15,16,17]. Nonetheless, the relations of light PA (LPA) in parents and young children have been explored in only a few studies, and therefore, it needs to be addressed in future research [7,12,13,15,16,17].

Similar significant positive relationships were also found between parents and their children in SB [4,14,15]. These associations were especially notable in mother–child dyads [4,14,15]. Yet, some discrepancies concerning weekdays vs. weekend days were documented across various studies [15,18,19,20]. Parental SB was significantly correlated with children’s SB consistently for weekdays and weekends [15] in some studies, whereas other research papers indicated higher parent–child associations in SB on weekends [18,19,20].

Given the established evidence highlighting the parent–child association in PA and SB [7,9,14,15], it is crucial to investigate the parental influence on children’s PA and SB. This examination has potential for several outcomes: (1) encouraging parents to adopt healthier behaviours, thereby serving as better role models for their children; (2) ensuring that children develop healthy habits during childhood, which may persist into adolescence and adulthood; and (3) providing a foundation for the development of effective interventions aimed at promoting healthier lifestyles. Furthermore, to develop targeted interventions effectively, research needs to incorporate data on gender and age disparities alongside socioeconomic and educational variables.

Based on the review article by Petersen [7], most studies focusing on the parent–child association in PA and SB originated in North America (n = 19), while others were carried out in Europe (n = 12), Australia (n = 6), New Zealand (n = 1), or China (n = 1) [7]. Only a small number of studies with at least a moderate sample size (n ≥ 200 parent–child dyads) monitored the parent–child association in PA/SB using objective measures in Europe (mostly in the UK) [12,21,22,23]. A few studies examined parent–child PA associations in Czech preschoolers/school-aged children using pedometers or questionnaires [24,25,26]. However, the use of an accelerometer is a preferred tool to measure PA in parents and children due to the lower risk of bias compared with pedometers [7]. Additionally, current research using objective measures often tends to focus more on older children and adolescents than on preschoolers and younger school-aged children [27]. No previous study has explored the association between parent and preschool-aged child PA and SB measured by accelerometers in Central Europe. 

Therefore, the aims of this study were to describe movement behaviours in Czech children aged 3–10 years and their parents and to analyse both mother–child and father–child interactions across all intensities of PA and SB. Furthermore, we established the following research questions: (1) How do boys and girls differ in their engagement in SB, LPA, MPA, VPA, MVPA and total PA?, (2) Are there significant differences in SB, LPA, MPA, MVPA, VPA and total PA between mothers and fathers?, (3) Are there mother–daughter (or son) and father–daughter (or son) associations in SB and different PA intensity levels (LPA, MPA, MVPA, VPA, and total PA)?, and (4) What factors are associated with children’s compliance in meeting the recommended levels of PA?

## 2. Materials and Methods

### 2.1. Participants and Selection

The selection of schools and kindergartens for the study was conducted using a two-round stratified random sampling method. In the first round, regions within the Czech Republic were randomly selected to ensure broad representation, with more than half of the regions included (8 out of 14 regions). In the second round, from the selected regions, random schools and kindergartens were contacted. The first schools and kindergartens that agreed to participate were included in the final sample, resulting in a total of 36 schools being part of the study. Of these, 36 institutions (77%) agreed to participate in the research. We reached out to 860 families across the following regions: Hradec Králové, Karlovy Vary, Liberec, Moravian-Silesian, Olomouc, Pardubice, South Bohemian, and Zlín. The exclusion criteria were children outside the age range of 3–10 years and those with any disease affecting their movement behaviour. Written consent was obtained from 552 families, each including at least one child and one parent. Out of 502 families that started the measurement procedure, we collected valid PA and SB data from ActiGraph measurements for 381 families (76%), which included 337 mothers, 256 fathers, 190 daughters, and 191 sons.

All of the participants followed a mandatory daily school/job routine. The data were collected during the spring and autumn of 2022 and the spring of 2023 [28]. This study was approved by the Ethical Committee of the Faculty of Physical Culture, Palacký University Olomouc. The children’s parents, their teachers, and school management representatives were informed in detail about the design of the survey including its objectives, the safety of the devices, the duration of monitoring, and the option to withdraw. The participants who agreed to participate in the research received individually set accelerometers with instructions on how to wear the device and the family logbook. All children and their parents participated in the study voluntarily and were free to withdraw at any time, either actively (verbally or in writing) or passively (by not wearing the device, handing in unused accelerometers, or submitting unfilled recording sheets). Printed graphical feedback on movement behaviours was provided to all study participants, including both children and parents, after completing the monitoring. Additionally, as a reward for their participation, the children received crayons. All materials were delivered to the families in sealed envelopes, and it was up to the parents to decide if and how they would communicate the results to their child.

### 2.2. Assessment of Physical Activity and Sedentary Behaviour

Different intensity levels of PA and SB were monitored using wGT3X-BT and GT9X Link ActiGraph accelerometers (ActiGraph, Pensacola, FL, USA). All participants were instructed to continuously wear an accelerometer on their non-dominant wrist for 24 h a day over a period of 7 consecutive days, except during activities such as swimming/diving and sauna use. Parents were provided with the GT9X Link model, while children were outfitted with the wGT3X-BT model. In addition, each accelerometer was labelled with a unique code corresponding to each family member to minimize the probability of miss-wearing. Simultaneously, parents kept a 7-day log that included information about the child’s waking times and bedtimes, PA, commuting to kindergarten/school, etc. [28]. 

The accelerometers were initialized to collect raw data with a 100 Hz sampling frequency, and after the data collection period, the data were downloaded using ActiLife software version 6.13.5. The raw data were processed using the open-source R package GGIR v3.0-3, applying Hildebrand’s age-specific ENMO thresholds for SB, LPA, MPA, and VPA [29,30]. Daily duration spent in SB and each PA intensity were calculated in min per day for all participants. Participants with valid data from at least 3 weekdays and 1 weekend day (defined as wear time at least 16 h/day) were included in the analyses.

### 2.3. Covariates

The anthropometric characteristics of the participants were compiled prior to the monitoring period for each participant. One week before the beginning of the monitoring, parents or guardians were requested to record their own and their children’s body height and weight, with accuracy to 0.5 cm and 0.1 kg, respectively, in the informed consent form. Proxy-reported height and weight of children, along with the derived body mass index (BMI), were validated against direct anthropometric measurements in children aged 6 to 18 years [31,32]. The BMI of the children was calculated using the reported height and weight, and the results were expressed as z-scores based on the sex- and age-specific WHO reference data. Children with a BMI z-score > 1 standard deviation (SD) and ≤2 SDs were classified as overweight, while those with a BMI z-score > 2 SDs were classified as obese [33,34]. Obese, overweight, and normal body mass in adult parents were determined according to BMI values. Overweight or obesity in parents represented BMI from 25 kg/m^2^ to 29.9 kg/m^2^, or greater than or equal to 30 kg/m^2^, respectively [35].

Family Affluence Scale (FAS) was included as covariates in our logistic regression analyses. Six questions were included to determine FAS: having one’s own bedroom (No = 0; Yes = 1), number of computers (None = 0; One = 1; Two = 2; Three or more = 3), car ownership (No = 0; One = 1; Two or more = 2), family holidays in the past year (Never = 0; Once = 1; Twice = 2; Three or more times = 3) dishwasher ownership (No = 0; Yes = 1) and number of bathrooms (None = 0; One = 1; Two = 2; Three or more = 3). The response codes to these items were summed and treated as a composite sum score. This score was then converted into a ridit (fractional rank) score ranging from 0 to 1, which could be used, due to its standardized form, for international comparisons of the results. Respondents were then divided into 20:60:20 ratio groups representing low, medium and high socioeconomic status [36].

Parents were asked about the highest level of education attained, with possible answers: no education, primary school, secondary school without matriculation, secondary school with matriculation, higher vocational school, university (bachelor’s degree), or university (master’s degree and above). This variable entered the logistic regression analysis as a binomial variable, with a score of 0 (high school with high school diploma and less education) and 1 (college and higher education).

The chronological age was calculated from the date of birth until the first monitored day. Age of children was entered in the logistic regression analysis as covariates with two categories: younger (3–5.9 years) and older children (6–10 years).

### 2.4. Statistical Analysis

The data were statistically analysed using SPSS v 26.0 software (IBM SPSS, Inc., Chicago, IL, USA). The Shapiro–Wilk test indicated that data for the selected variables were not normally distributed. Therefore, non-parametric statistical methods were applied. To assess differences between boys and girls, or mothers and fathers, the Mann–Whitney U test was used. Bivariate correlations (Spearman’s rho) were conducted to examine the parent (mothers, fathers)–child (daughters, sons) association for the following variables: LPA, MPA, VPA, MVPA, total PA, and SB (all in minutes per day).

Quantifications of achieving PA recommendations were based on World Health Organization guidelines and Canadian 24 movement guidelines [37,38,39] and meeting the PA recommendation was defined as participating in at least 180 min/day of total PA and at least 60 min/day of MVPA in children aged 3 or 4 years and accumulating at least 60 min/day of MVPA in children aged 5 years and older. This variable was set as a dependent binominal variable in the logistic regression analysis (0 = not meeting the recommended PA; 1 = meeting the recommended PA).

Logistic regression (the backward LR method) was used to examine the achievement of the current PA recommendations in children. The tested model included the following independent child and parental variables: children’s daily sedentary time (continuous), children’s gender (boys vs. girls), children’s age category (3–5.9 y vs. 6–10 y), children’s weight status (overweight/obese vs. normal body weight), FAS (low, moderate, high), mothers’/fathers’ weight status (overweight/obese vs. normal body weight), mothers’/fathers’ daily sedentary time (continuous); mothers’/fathers’ VPA (continuous); mothers’/fathers’ education (high school with high school diploma and less education vs. college and higher/university education). Statistical significance was set at alpha level 0.05.

## 3. Results

### 3.1. Gender Differences in Physical Activity Intensity Levels and Sedentary Behaviours

**Figure 1:**
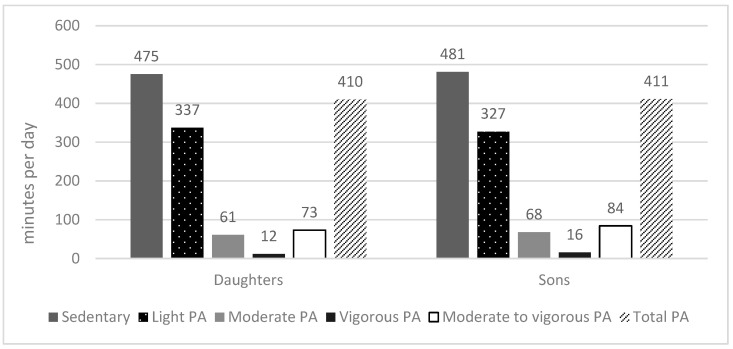
Time spent in different PA intensities and SB in daughters and sons.

As shown in Figure 1, boys and girls were observed to spend a significant portion of the day in SB (boys 481 ± 76 min per day, girls 475 ± 74 min per day). In contrast, boys (M = 16 ± 11 min per day) and girls (M = 12 ± 7 min per day) were found to spend the least amount of the day in VPA. Based on the results of the Mann–Whitney U test, girls reported significantly more LPA than boys (*p* = 0.009), whereas boys were significantly more active in MPA (*p* = 0.026), VPA (*p* = 0.004) and MVPA (*p* = 0.011). However, no statistically significant differences were found between boys and girls in SB (*p* = 0.261) and total PA (*p* = 0.664).

**Figure 2:**
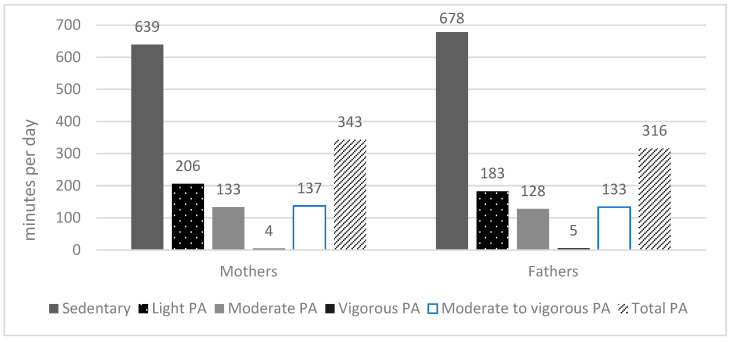
Time spent in different PA intensities and SB in mothers and fathers.

As presented in Figure 2, both mothers and fathers spent the majority of their day sedentary (mothers 639 ± 82 min per day, fathers 678 ± 98 min per day). In addition, mothers (4 ± 6 min per day) and fathers (5 ± 7 min per day) devoted the smallest portion of the day to VPA. There were no significant differences in MPA (*p* = 0.310), VPA (*p* = 0.085), or MVPA (*p* = 0.436) between mothers and fathers (Figure 2). However, mothers were shown to spend significantly less time in SB (*p* < 0.001), and significantly more time in LPA (*p* < 0.001) and total PA (*p* < 0.001), compared to fathers.

### 3.2. Association between Parents’ and Children’s Time Spent Sedentary, and Time Spent in Different Physical Activity Intensities

As indicated in Table 1, the analysis of the associations between parents and children pointed to closer significant associations between mothers and daughters in sedentary time, LPA, VPA and total PA than in father–daughter associations. The mother–son association was stronger in VPA compared to father–son pairs. On the contrary, there was a slightly stronger significant association between fathers and daughters in MPA and MVPA. In addition, a closer significant correlation in total PA was documented in fathers and sons than in mothers and sons. For the other variables (SB, LPA, MPA, and MVPA), similar associations were found in both mother–son and father–son dyads. (Table 1).

### 3.3. Logistic Regression Analyses to Achieve Moderate to Vigorous PA Recommendation

The presented logistic regression model for fulfilling the children’s PA recommendation included children’s and parents’ weight status, children’s and parents’ daily sedentary time, parents’ education, parents’ VPA, children’s gender, and age category. Children whose mothers engaged in more VPA were significantly more likely to comply with the PA recommendations. In both mother and father models, girls were significantly less likely to meet the PA recommendations than boys (Table 2). Additionally, children who spent more time sedentary were significantly less likely to fulfil the PA recommendation. Older children aged 6–10 years were significantly (approximately three times) more likely to meet the PA recommendation compared to younger children aged 3–5.9 years.

## 4. Discussion

To our knowledge, this is the first study addressing parent–child relations across PA intensity levels and in SB using accelerometer-based measurements in gender-differentiated dyads of parents and children from Central Europe.

The results of this study revealed that children and parents spent most of the day sedentary rather than being active. These findings are in line with worldwide data [40,41,42]. More than 50% of pre-schoolers and 40% of school-aged children were observed to spend the majority of the day in SB [40,41]. The PA guidelines were met by only 32.7% of pre-schoolers [3], and 22.8% of parents [42].

Concerning gender differences among children, our results showed no significant difference in total PA. This finding was not supported by the current literature, which indicated that more boys than girls participated in total PA [24,42,43,44]. This discrepancy might be explained in part by the methodological differences, i.e., the measuring tools used or the quantification of PA. Even though there was no variation in total PA, our findings observed that girls engaged in more LPA, while boys engaged in more MVP, MVPA, and VPA compared to the opposite gender. Moreover, in contrast with our outcomes, according to a systematic review (2019), girls spend more time sitting than boys [40]. In kindergarten, which almost 88% of Czech children attend on a regular basis [45], the PA-related activities were the same regardless of gender. This similarity might have caused the equal amount of time spent in SB and total PA in boys and girls.

With respect to parental gender differences, our study revealed that mothers spent significantly less time in SB and more time in LPA and total PA compared to fathers. Unlike our discovery, other studies observed no gender variance in PA between Czech parents [24,25]. The measuring tools (pedometer vs. accelerometer) used and changes in parent’s active behaviour might be a potential reason for this difference. Another possible explanation for higher PA in mothers could be related to their specific role within the family, such as caregiving for children, involvement in household chores, shopping, etc. [46,47]. These responsibilities may lead to higher levels of PA compared to fathers [46,47].

Our findings also indicated a positive parent–child association only in some PA levels and SB (mother–daughter SB, LPA, VPA and total PA; mother–son VPA; father–daughter MPA and MVPA; father–son total PA). The majority of previous studies examining the association between PA (mostly MVPA and total PA) of parents and children across various gender and age combinations found a positive correlation between parent (mother and/or father) and child [7,9,10,24]. This is consistent with our outcomes in mother–daughter (total PA) and father–son (total PA and MVPA) dyads. Mixed results are available in a limited number of studies that addressed parent–child associations in LPA and VPA intensities [12,13,16,17,48,49]. Most studies on the correlation between parent and child LPA showed a positive association in parent/mother child dyads [12,16,48,49] (consistent with our results only in mother–daughter dyads), whereas a few studies revealed no associations [13,17]. Limited attention has been devoted to monitoring VPA parent–child associations [49]. Unlike our discovery, which indicated a close mother–son/daughter association in VPA, this association was not shown to be significant in one study that addressed it [49]. Mixed findings of LPA and the lack of data on VPA parent–child associations indicate the necessity for further investigation.

Related to gender-differentiated resemblances between parent and child PA, our findings pointed out that in total PA, mothers tend to have more influence on their daughters. Fathers, on the other hand, were more linked with their sons. This interconnection was supported by previous research that detected a similar trend in same gender pairs regarding positive PA [7,9,50,51,52] and SB associations [12,53]. In the family, mothers and fathers differed in their parenting style and behaviour modelling of their children [46,47,52,54]. Especially for young children, mothers are more inclined to be more involved in childcare, housekeeping and caregiving than fathers [46,47,55]. This greater involvement could explain the closer link found more frequently between mother and child (especially daughter) activity habits. However, it was documented that parents had different PA parenting practices, and while mothers tend to have more influence on their children’s SB, screen time and diet, fathers had more of an effect on shaping their children’s (especially sons’) PA (MVPA and VPA) [52,54]. At least one positive association between father and child PA was observed in previous studies [10,53,56,57] (including ours). Nonetheless, due to the limited number of male participants in some studies [53,56], this might have caused a non-significant relationship between parent and child PA and SB. Therefore, it is difficult to draw conclusions about father–child interrelations in PA of different intensity levels and SB, which should be further monitored.

We also found that older children (6–10 years old), compared to those aged 3–6 years, had a higher chance of meeting the PA recommendations. This could be related to their already acquired experience in participating in organized sport activities through school or sport clubs at a younger age. Still, some inconsistencies were noticed, as one study showed the PA interconnection was strongest between mothers and younger children (aged 4–7.9 years) [24], and others pointed out the strongest link between parents and 10–12-year-olds [51]. Since it may be possible that the strength of this correlation could influence children’s fulfilment of the PA guidelines, further investigation is warranted.

### Strengths and Limitations

The strengths of this study include the use of accelerometer-based measurements for SB and PA in children, mothers, and fathers; a large sample size of Czech families; a high response rate; and numerous mother–child and father–child dyads. However, it also presents some limitations that need to be acknowledged: (1) the cross-sectional design does not allow for inferring causality; (2) there is an absence of analysis on the temporal association of PA between parents and children, including whether activities were synchronized and executed together; (3) it is possible that more active families were more likely to agree to participate in the study; (4) participants may have been subjected to the “measurement effect”, resulting in modifications of their behaviours during the measurement process.

## 5. Conclusions

This study revealed that both children and parents were predominantly sedentary throughout the day as opposed to engaging in PA. Regarding disparities between genders, no significant difference was found in total PA in children; however, mothers allocated significantly less time to SB and more time to LPA and total PA compared to fathers. Additionally, our findings suggested positive parent–child associations only in some PA levels and SB. It was also found that mothers had closer relationships with their children in most levels of PA compared to fathers. Finally, factors associated with children meeting the PA guidelines included child’s male gender, older age (6–10 years), engaging in less SB, and having mothers who participated in VPA. The evidence from this study highlights the importance of promoting healthy PA and reducing SB within the entire family, not just among parents or children. This approach may also help reduce gender disparities. Furthermore, future strategies to improve children’s daily compliance with PA guidelines might consider including mother–child dyads in exercise programs of moderate to high intensity levels (i.e., jumping or tag games) [11,58]. Further research should investigate mother–child and father–child associations in LPA and VPA, with a particular focus on father–child dyads.

## Figures and Tables

**Table 1:** Bivariate correlations between mothers’, fathers’, and children’s (daughters, sons) minutes per day of different PA intensities and SB (Spearman’s rho).

	**Daughters**					
Variables	SB	LPA	MPA	VPA	MVPA	Total PA
Mothers	0.171 *	0.283 ***	0.282 ***	0.163 *	0.288 ***	0.337 ***
(n = 167 pairs)						
Fathers	−0.011	0.131	0.302 ***	0.100	0.299 ***	0.265 **
(n = 125 pairs)						
	**Sons**					
Variables	SB	LPA	MPA	VPA	MVPA	Total PA
Mothers	0.221 **	0.241 **	0.276 ***	0.298 ***	0.283 ***	0.246 ***
(n = 170 pairs)						
Fathers	0.221 *	0.214 *	0.257 **	0.093	0.241 **	0.333 ***
(n = 131 pairs)						

Spearman’s rho of the corresponding variables; * *p* < 0.05; ** *p* < 0.01; *** *p* < 0.001; sedentary behaviour (SB), light physical activity (LPA), moderate physical activity (MPA), vigorous physical activity (VPA), moderate to vigorous physical activity (MVPA), and physical activity (PA).

**Table 2:** Logistic regression analyses to achieve children’s PA recommendation.

	Mother in Model			Father in Model		
	n	OR	95% CI	n	OR	95% CI
Parent: **Vigorous PA**						
(continuous)	315	1.069 *	1.01–1.14		X	
*Children:*						
**Gender**						
Boys	160	Ref.		121	Ref.	
Girls	155	0.488 *	0.27–0.89	109	0.444 *	0.22–0.91
**Age category**						
3–5.9 years	121	Ref.		88	Ref.	
6–10 years	194	2.883 **	1.54–5.40	142	3.035 **	0.29–0.82
**Sedentary behaviour**						
(continuous)	315	0.978 ***	0.97–0.98	230	0.978 ***	0.97–0.99
Nagelkerke R^2^		0.419			0.405	

Logistic regression analyses to achieve children’s PA recommendation, backward stepwise method (likelihood ratio); * *p* < 0.05; ** *p* < 0.01; *** *p* < 0.001; OR odds ratio; Ref. reference category; CI confidence interval, X variable excluded from the model; Nonsignificant variables excluded from the model: children’s weight status, FAS, mothers’/fathers’ weight status, mothers’/fathers’ SB, mothers’/fathers’ education.

## Data Availability

The datasets presented in this article are not readily available because this study is part of a longitudinal research project. The baseline phase data are subject to privacy and ethical restrictions as participants in this study have signed consent forms stipulating that individual data will not be made publicly available until five years after the completion of the follow-up phase.
